# Supplementary material for: Bioinformatic Analyses of Broad H3K79me2 Domains in Different Leukemia Cell Line Data Sets
Source: Cells. 2022 Sep 10;11(18):2830. doi: 10.3390/cells11182830 (PMC9496709; doi:10.3390/cells11182830)
Supplement: Supplementary file 1 [file cells-11-02830-s001.zip › Supplementary files/Supplementary Materials (Figures).pdf]

Supplementary Materials

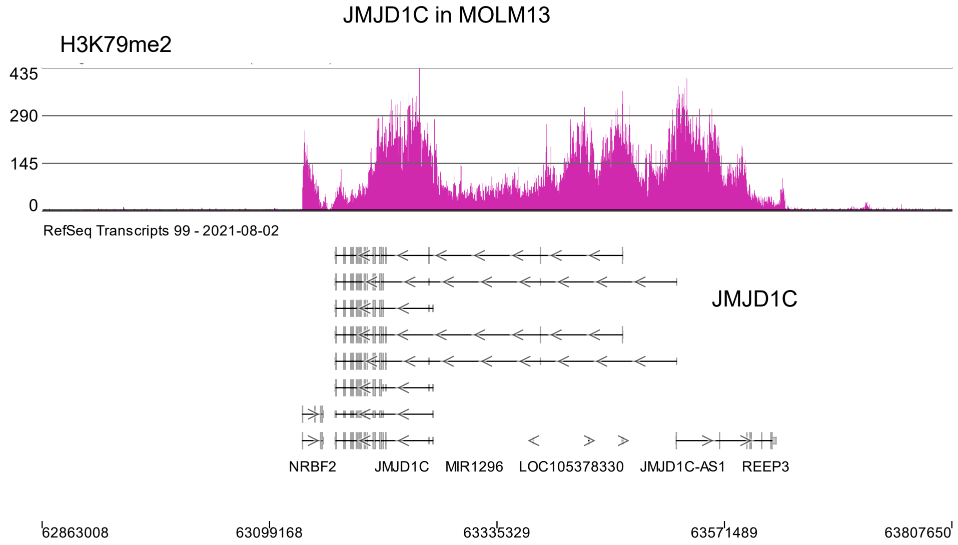

Figure S1. H3K79me2 ChIP-Seq track from *JMJD1C* gene in MOLM-13 cells.

Figure S2A

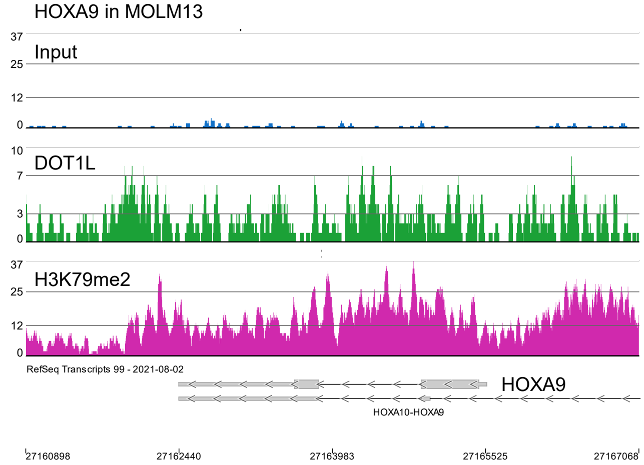

Figure S2B

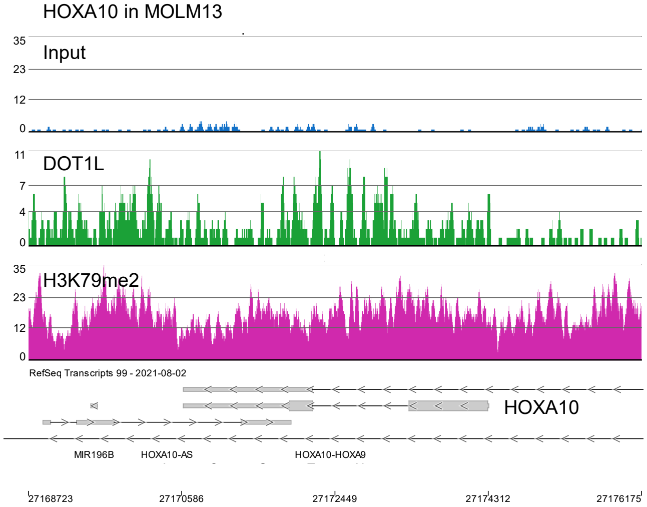

Figure S2C

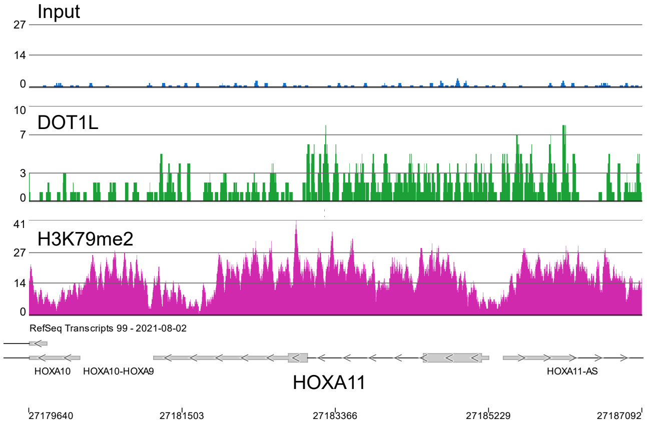

Figure S2. H3K79me2 ChIP-Seq tracks for *HOXA9*, *HOXA10* and *HOXA11* genes in MOLM-13 cells.

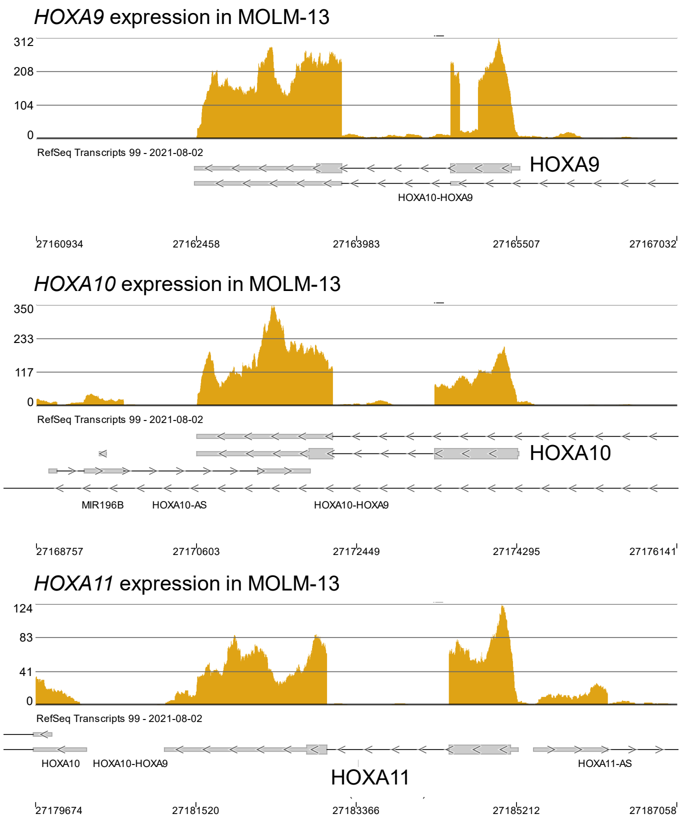

Figure S3. RNA-Seq tracks for *HOXA9*, *HOXA10* and *HOXA11* genes in MOLM-13 cells.
