# Supplementary material for: Bioinformatic Analyses of Broad H3K79me2 Domains in Different Leukemia Cell Line Data Sets
Source: Cells. 2022 Sep 10;11(18):2830. doi: 10.3390/cells11182830 (PMC9496709; doi:10.3390/cells11182830)
Supplement: Supplementary file 1 [file cells-11-02830-s001.zip › Supplementary files/Table S2.pdf]

Table S2: Genes in *HOXA9* gene cluster

Gene	TPM	Functional role	**Reference** (PMID)
HOXA1 (Homeobox A1)	15.2	• Encodes transcription factor which is needed for controlling gene expression, morphogenesis, and differentiation<br>• Play a role in oncogenesis via TNF/NF-κB<br>• Contributes to cancer progression	27382069,23088713, 22498108, 22864671, 23435427, 26791264, 32561925, 31426381, 16909099, 15084694
HOXA2 (Homeobox A2)	0.79	• Encodes transcription factor which is needed for controlling gene expression, morphogenesis, and differentiation<br>• Involved in homeostasis<br>• Play a part in the embryonic development via Wnt–β-catenin<br>• Contributes to cancer progression	32436804, 32649979, 1358459, 22223247
HOXA3 (Homeobox A3)	0.67	• Encodes transcription factor which is needed for controlling gene expression, morphogenesis, and differentiation<br>• Involved in organ development<br>• Associated with the Colon Cancer progression through EGFR/Ras/Raf/MEK/ERK<br>• Contributes to cancer progression	15714286, 11784044, 30604847, 29073728
HOXA4 (Homeobox A4)	2.8	• Encodes transcription factor which is needed for controlling gene expression, morphogenesis, and differentiation<br>• Contributes to cancer progression	17785556, 29700285, 16525643, 23980595
HOXA5 (Homeobox A5)	14.5	• Encodes transcription factor which is needed for controlling gene expression, morphogenesis, and differentiation<br>• Play a role in Adipose Differentiation via PKA/HSL pathway<br>• Contributes to cancer progression	35219772, 33136093, 33223227
HOXA6 (Homeobox A6)	3.3	• Encodes transcription factor which is needed for controlling gene expression, morphogenesis, and differentiation<br>• Play a role in Renal Cell Carcinoma via PI3K/Akt<br>• Contributes to cancer progression	31081053, 19157684, 26870170
HOXA7 (Homeobox A7)	27.3	• Encodes transcription factor which is needed for controlling gene expression, morphogenesis, and differentiation<br>• Associated with the progression of cancers<br>• Involved in MLL-mediated transformation	17959889, 19424076, 11070089, 21626505
HOXA9 (Homeobox A9)	50.5	• Encodes transcription factor which is needed for controlling gene expression, morphogenesis, and differentiation<br>• Play a role in leukemia via Wnt–β-catenin and JAK/STAT<br>• Play a part in pyroptosis of human trophoblasts via CMKLR1/AMPK/TXNIP/NLRP3 signaling<br>• Contributes to cancer progression	20339075, 34461109, 29496663
HOXA10 (Homeobox A10)	67.5	• Encodes transcription factor which is needed for controlling gene expression, morphogenesis, and differentiation<br>• Play a role in infertility and viability of the embryo<br>• Associated with the progression of cancers via TGFB2/Smad/METTL3, Wnt/β-catenin, PTEN/AKT/PI3K, and NF-κB signaling pathways	20821045, 35550944, 33563300 32299243, 25120782, 35553652 34293937, 32571260, 34496932 30614022
HOXA11 (Homeobox A9)	56.3	• Encodes transcription factor which is needed for controlling gene expression, morphogenesis, and differentiation<br>• Play a part in endometrial carcinoma via PTEN/AKT signaling pathway<br>• Involved in uterine development and female fertility<br>• "Double-agent" gene (proto-oncogenes and tumor-suppressor function)	33515421, 20695189, 12050232 29914539, 28423531, 35430506

HOXA11-AS (HOXA11 antisense RNA)	15.9	• Produces lncRNA<br>• Play a part in embryo implantation, endometrial development, and inflammation<br>• Associated with the progression of cancers via PI3K/AKT, WNT1/β-catenin, JAK-STAT	9745033, 26054679, 30402857, 31099097
HOXA13 (Homeobox A13)	28.5	• Encodes transcription factor which is needed for controlling gene expression, morphogenesis, and differentiation<br>• Involved in Colon Cancer progression via Wnt/β-Catenin<br>• Contributes to cancer progression	32822724, 29678634
HOXA-AS2 (HOXA cluster antisense RNA 2)	0.16	• Produces lncRNA<br>• Play a role in acute myeloid leukemia via SOX4/PI3K/AKT<br>• Associated with the progression of cancers via EGFR/Ras/Raf/MEK/ERK, Notch, and NF-κB	34076871, 30658190, 32369230 30551418
HOXA-AS3 (HOXA cluster antisense RNA 3)	0.27	• Produces lncRNAs<br>• Control endothelium inflammation via NF-κB, MEK/ERK<br>• Contributes to cancer progression	31285272, 30963785
HOTTIP (HOXA distal transcript antisense RNA)	0.47	• produces lncRNAs (antisense to the HOXA gene cluster)<br>• Associated with progression of cancers via NF-κB, MEK/ERK, Wnt/β-catenin<br>• "Double-agent" gene (proto-oncogenes and tumor-suppressor function)	35210436, 33000231, 32307830, 31002141
HOTAIRM (HOXA transcript antisense RNA, myeloid-specific 1)	4.82	• Produces lncRNA, which is specific for maturing myeloid cells<br>• Control the cell cycle progression during the maturation of myeloid leukemia cells<br>• Play a role in leukemia via the Wnt/β-catenin<br>• Involved in tumorigenesis via PFKP, NF-κB, JNK/AP-1, and JNK/MAPK/ERK	29164055, 31904363, 33404645, 31512358, 30551410
HOXA10-AS (HOXA10 antisense RNA)	13.6	• Produces lncRNAs (transcribed from the antisense strand of HOXA10 gene<br>• Involved in Lung Adenocarcinoma through the Wnt/β-catenin<br>• Contributes to cancer progression	29729275, 30132568, 31867596
